# Multi-scale numerical simulations of thermal expansion properties of CNT-reinforced nanocomposites

**DOI:** 10.1186/1556-276X-8-15

**Published:** 2013-01-07

**Authors:** Alamusi, Ning Hu, Jianhui Qiu, Yuan Li, Christiana Chang, Satoshi Atobe, Hisao Fukunaga, Yaolu Liu, Huiming Ning, Liangke Wu, Jinhua Li, Weifeng Yuan, Tomonori Watanabe, Cheng Yan, Yajun Zhang

**Affiliations:** 1 Department of Mechanical Engineering, Chiba University, Yayoi-cho 1-33, Inage-ku, Chiba, 263-8522, Japan; 2 School of Manufacturing Science and Engineering, Southwest University of Science and Technology, 59 Qinglong Road, Mianyang, 621010, People's Republic of China; 3 Department of Machine Intelligence and Systems Engineering, Akita Prefectural University, Akita, 015-0055, Japan; 4 Department of Nanomechanics, Tohoku University, 6-6-01 Aramaki-Aza-Aoba, Aoba-ku, Sendai, 980-8579, Japan; 5 Department of Mechanical Engineering, University of Houston, 4800 Calhoun Road, Houston, TX, 77004, USA; 6 Department of Aerospace Engineering, Tohoku University, 6-6-01 Aramaki-Aza-Aoba, Aoba-ku, Sendai, 980-8579, Japan; 7 School of Engineering Systems, Queensland University of Technology, 2 George Street, GPO Box 2434, Brisbane, 4001, Australia; 8 College of Mechanical and Electrical Engineering, Beijing University of Chemical Technology, Beijing, 100029, People's Republic of China

**Keywords:** Polymer-matrix composites (PMC), Thermal properties, Numerical analysis, Carbon nanotube (CNT)

## Abstract

In this work, the thermal expansion properties of carbon nanotube (CNT)-reinforced nanocomposites with CNT content ranging from 1 to 15 wt% were evaluated using a multi-scale numerical approach, in which the effects of two parameters, i.e., temperature and CNT content, were investigated extensively. For all CNT contents, the obtained results clearly revealed that within a wide low-temperature range (30°C ~ 62°C), thermal contraction is observed, while thermal expansion occurs in a high-temperature range (62°C ~ 120°C). It was found that at any specified CNT content, the thermal expansion properties vary with temperature - as temperature increases, the thermal expansion rate increases linearly. However, at a specified temperature, the absolute value of the thermal expansion rate decreases nonlinearly as the CNT content increases. Moreover, the results provided by the present multi-scale numerical model were in good agreement with those obtained from the corresponding theoretical analyses and experimental measurements in this work, which indicates that this multi-scale numerical approach provides a powerful tool to evaluate the thermal expansion properties of any type of CNT/polymer nanocomposites and therefore promotes the understanding on the thermal behaviors of CNT/polymer nanocomposites for their applications in temperature sensors, nanoelectronics devices, etc.

## Background

As technology and modern industry has developed, reinforced composite materials, such as particle- or short-fiber-reinforced composites and long-fiber-reinforced or sandwich laminates, have been widely applied in the aerospace, construction, transportation, machinery, chemical, and other industries. In recent years, as a representative of new engineering materials, carbon nanotube (CNT) at nanoscale has shown superior mechanical, electrical, and thermal properties, as well as low density and high aspect ratio, which make it an ideal choice for composite reinforcement. CNT-reinforced nanocomposite is a multi-phase material, and its external macro-physical properties strongly depend on the properties of its constituents and complex internal microstructure. Experimental evaluation requires large amounts of material samples and a large testing work load, giving simulation of the physical properties of nanocomposites important engineering significance.

There has been extensive research on the mechanical, thermal, and electrical properties of CNT-reinforced nanocomposites. For instance, the thermal properties [1-3] and electrical properties of CNT-reinforced nanocomposites [4,5] have been explored experimentally in some previous studies. Moreover, due to the complexity and variations of the CNT-reinforced composite microstructure, theoretical analyses and numerical simulation methods are common strategies to estimate composite physical properties. For instance, diffusion and thermal expansion coefficients of CNT-reinforced nanocomposites have been studied through micromechanics models without sufficient atomic scale information [6] or molecular dynamics (MD) models with very high computational cost and complexity [7].

In recent years, to deal with the remarkable scale difference in CNT-reinforced nanocomposites, multi-scale modeling has been widely used for predicting the mechanical properties [8], electrical properties [9], and thermal conductivity [10] of the CNT-reinforced nanocomposites. However, to the best knowledge of the present authors, there has been no report on the multi-scale modeling of thermal expansion properties of the CNT-reinforced nanocomposites to date. In this work, the thermal expansion properties of the CNT-reinforced nanocomposites, i.e., CNT/epoxy, were evaluated using a sequential multi-scale numerical model. The present study focused on the effects of two key parameters, i.e., temperature and CNT content, on the thermal expansion properties. Moreover, it was found that the results of the present multi-scale numerical model agree very well with those based on theoretical predictions and experimental measurements carried out in this work.

## Methods

**Figure 1:**
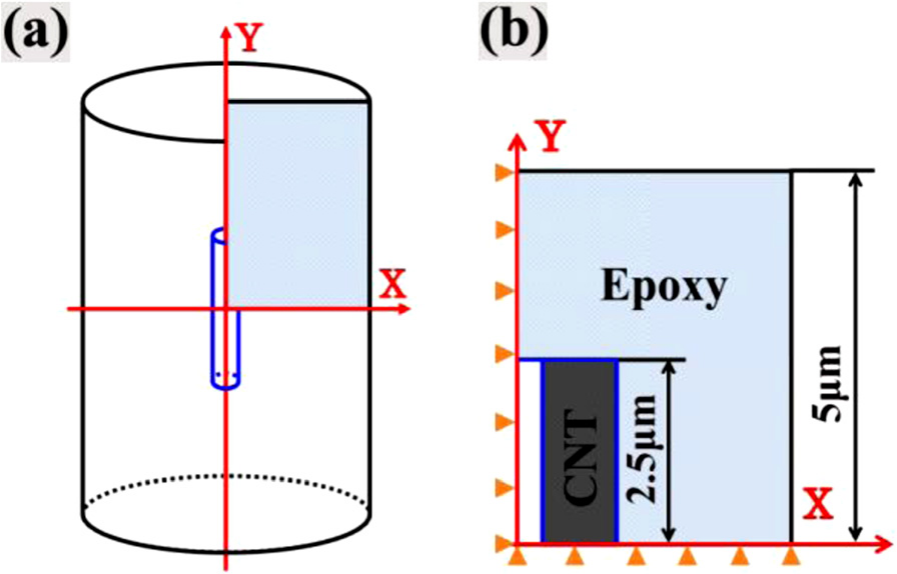
**Schematic of uni-directional numerical model. **(**a**) A cylindrical model (RVE). (**b**) Schematic of a quarter axisymmetric model.

**Figure 2:**
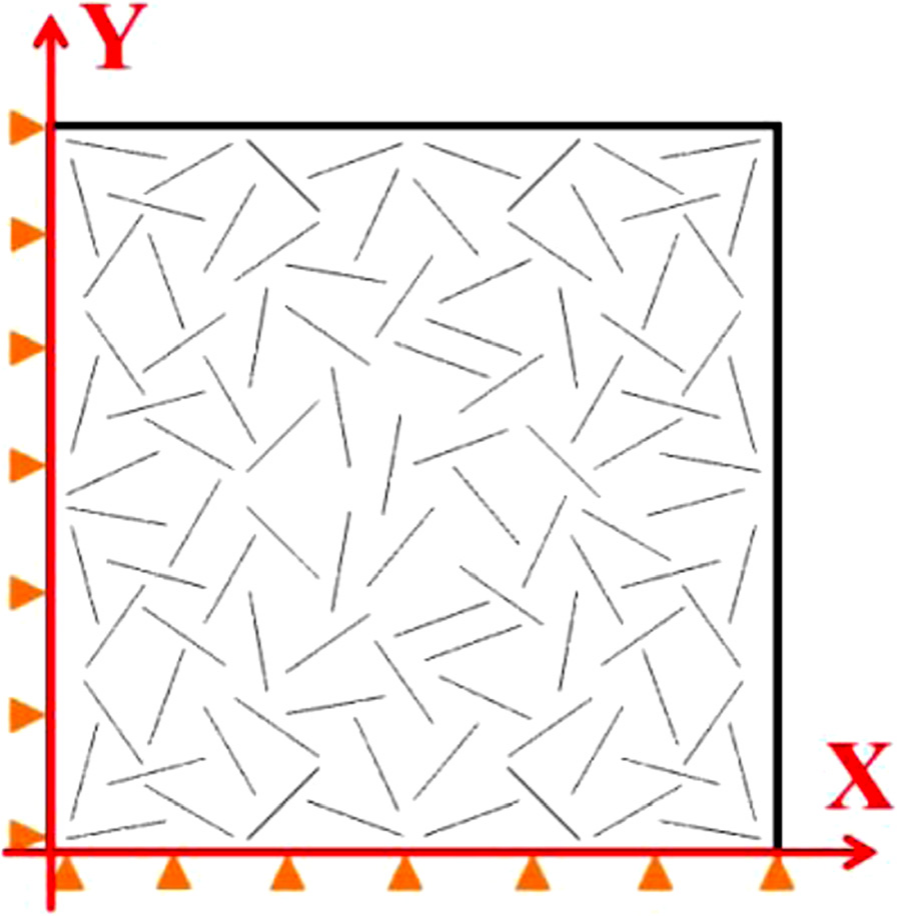
Schematic of multi-directional numerical model.

To investigate the thermal expansion properties of CNT-reinforced nanocomposites, numerical simulations based on a sequential multi-scale approach were conducted on two types of microstructural models, a uni-directional model in which CNTs were uni-directionally aligned within epoxy and a multi-directional model in which the CNTs were randomly oriented within the epoxy. For CNT-reinforced nanocomposites, uni-directionally aligned CNTs in matrix can be realized by applying electric [11] or magnetic fields during the curing process. The uni-directional model was constructed as a two-dimensional (2D) axisymmetric model (see Figure 1), and the multi-directional model was built up as a 2D plane strain unit cell model (see Figure 2). Note that to reduce the computational cost, an equivalence conversion principle [12,13] from three-dimensional (3D) modeling to 2D modeling for short-fiber-reinforced composites was used as a supporting evidence for the present 2D plane strain multi-directional model.

**Figure 3:**
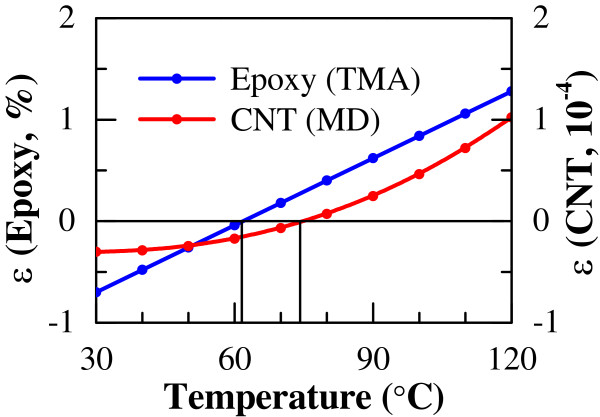
Thermal expansion rates of CNT and epoxy.

To construct the sequential multi-scale numerical model, we firstly used the axial thermal expansion properties of multi-walled carbon nanotube (MWCNT), which were obtained from extensive MD simulations at atomic scale in the authors' previous work [14]. Secondly, continuum mechanics-based microstructural models, i.e., the uni-directional and multi-directional ones, were built up based on the MWCNT's thermal expansion properties at atomic scale and the thermal expansion properties of epoxy obtained from experimental thermomechanical analysis (TMA) measurements in this work. The detailed description of experiments will be provided later. The thermal expansion rates *ε* of the present MWCNT and epoxy from 30°C to 120°C are shown in Figure 3. As shown in [14], the axial thermal expansion rate of MWCNT is dominated by MWCNT's inner walls. We modeled MWCNT's six innermost walls [14] to obtain the approximate axial thermal expansion rate of the present MWCNT in Figure 3.

In the uni-directional and multi-directional models used for the finite element analysis, the present multi-scale numerical simulations were conducted under the following conditions:

1. The CNT content of CNT/epoxy nanocomposites ranged from 1 to 15 wt%.

2. The length and diameters of the outmost and innermost walls of CNT were set as 5 μm, 50 nm, and 5.4 nm, respectively, which are in accordance with the experimental measurement using a transmission electron microscope [9,15]. The properties of MWCNT used in the present experiments are shown in Table 1.

**Table 1:** Properties of MWCNT

**Property**	**Value**
Fiber diameter (nm)	Average 50
Aspect ratio (−)	>100
Purity (%)	>99.5

3. We only considered the axial thermal expansion/contraction of MWCNT, and the radial thermal expansion/contraction was neglected since they are very small as identified in [14]. Therefore, CNT thermal expansion properties were orthotropic. Other properties of CNT were assumed to be isotropic, as well as those of epoxy. The detailed material properties in simulations are listed in Table 2.

**Table 2:** Material properties

**Property**	**CNT**	**Epoxy**
Density (g/cm^3^)	2.1	1.1
Young's modulus (GPa)	1,000	3.2
Poisson's ratio	0.1	0.34
Specific heat (mJ/g·K)	650	1,000
Thermal conductivity (W/mm·K)	6.7	2 × 10^−4^
CTE (K^−1^)	From Figure 3	From Figure 3

4. For the uni-directional model, simulations were conducted using a quarter of the cross section of a cylinder representative volume element (RVE) containing a CNT, i.e., an axisymmetrical model (see Figure 1). Under thermal loading, some forces along the radial direction were imposed on the nodes of the outmost lateral surface of the RVE and adjusted through an iterative procedure so that all points on the outmost lateral surface moved at the same distance in the radial direction to simulate the periodic conditions [16]. The length of the polymer was two times longer than that of the CNT in Figure 1, implying that the short CNTs are distributed evenly in both longitudinal and lateral directions in a matrix so that the RVE is the same for any CNT [16].

5. For the multi-directional model, there were randomly distributed 100 CNTs per model (see Figure 2). This model was built up under plane-strain conditions. The boundary conditions were applied at the two external edges which is similar to those for the uni-directional model above. In order to reflect the 3D characteristics of real nanocomposites, the volume fraction should be converted to the half of the real one [12,13]. Note that the number of the CNTs in this model, i.e., 100, was determined by some trial computations, such as testing of models containing 10, 25, and 50 CNTs. It was found that 100 is the minimum number, which can yield isotropic, convergent, and stable results. This number is just the same with that of holes for modeling the effective mechanical properties of a porous plate [17].

## Results and discussion

### Uni-directional models

**Figure 4:**
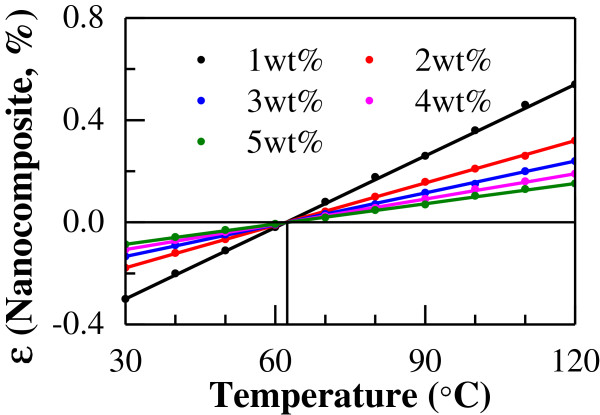
Thermal expansion rate of uni-directional CNT/epoxy nanocomposite by numerical simulation.

Firstly, we investigated the influences of temperature and CNT content on the thermal expansion properties of CNT/epoxy nanocomposites by varying the temperature from 30°C to 120°C and CNT content from 1 to 5 wt%. The thermal expansion properties vary with temperature, as shown in Figure 4. In this figure, the thermal expansion rate increases linearly as temperature increases for any loading of CNT. The temperature of zero thermal expansion rate (or no thermal expansion/contraction) of the CNT/epoxy nanocomposites is approximately 62°C, which is independent of CNT loading. Moreover, at a specified temperature, the absolute value of thermal expansion rate decreases with increasing content of CNT. The influence of the nonlinear thermal expansion rate of CNT (Figure 3) on that of the nanocomposites seems to be small due to very low CNT contents in Figure 4.

**Figure 5:**
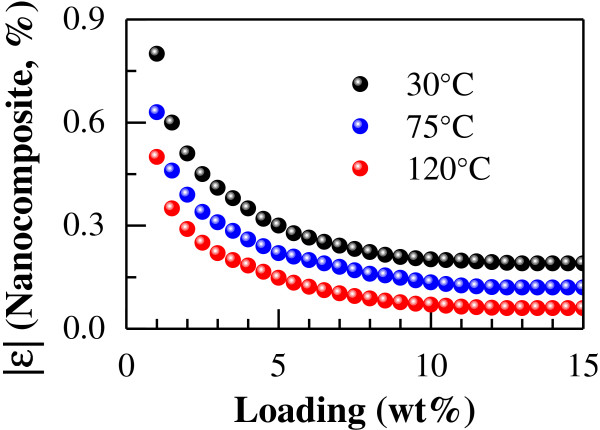
**Relationship between CNT content and absolute value of thermal expansion rate of uni-directional CNT/epoxy nanocomposite.** (Data of 30°C = Original data × (−2.5); data of 75°C = Original data × 8).

Although it is still a technical challenge to uniformly disperse CNTs for high loading, e.g., over 10 wt%, to numerically explore the thermal expansion properties in detail, the content of CNT was varied from 1 to 15 wt%, and the corresponding results are shown in Figure 5 with some artificial adjustments due to the big differences in various curves. From Figure 5, the thermal expansion rates vary nonlinearly with the content of CNT. In the range of 1 to 5 wt%, the change of thermal expansion rate is obvious. Beyond 5 wt%, the increase of CNT content within the temperature range (30°C ~ 120°C) results in the absolute values of the thermal expansion rate |*ε*| becoming gradually smaller and finally converging to a stable value when the CNT content reaches 10 wt%. Note that the thermal expansion rate is negative at 30°C.

### Multi-directional models

**Figure 6:**
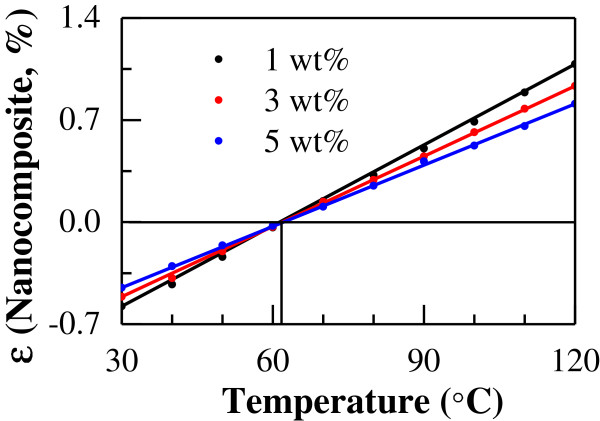
Thermal expansion rate of multi-directional CNT/epoxy nanocomposite by numerical simulation.

The ranges of temperature and CNT content in this case are identical to those mentioned above for the uni-directional models. The variation of thermal expansion properties of CNT/epoxy nanocomposites is shown in Figure 6 (CNT content from 1 to 5 wt%), in which the similar effects of temperature and CNT content are observed. In this figure, the thermal expansion rates increase linearly as the temperature increases for all CNT contents. The temperature at zero thermal expansion rate (or no thermal expansion/contraction) of the CNT/epoxy nanocomposites is approximately 62°C at any CNT loading, which is similar to that for the uni-directional model. With increasing content of CNT, the absolute value of thermal expansion rate decreases. Moreover, compared to the uni-directional nanocomposites (Figure 4), at high temperature, the difference in thermal expansion between low CNT content (1 wt%) and high CNT content (5 wt%) is much smaller in the multi-directional nanocomposites.

**Figure 7:**
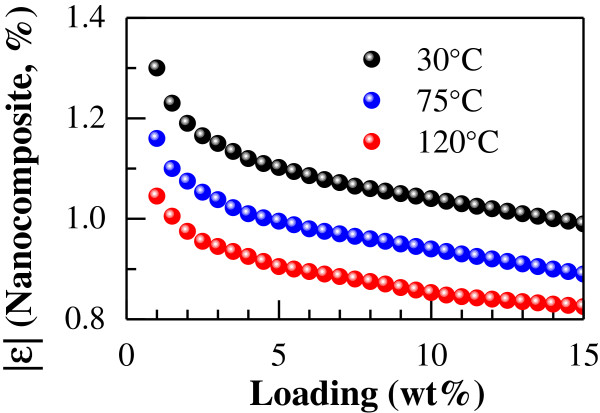
**Relationship between CNT content and absolute value of thermal expansion rate of multi-directional CNT/epoxy nanocomposite.** (Data of 30°C = Original data × (−2.5); data of 75°C = Original data × 8).

By varying the CNT content from 1 to 15 wt%, the obtained results are shown in Figure 7. In this figure, the thermal expansion rates vary nonlinearly with the CNT content. In the content range of 1 to 5 wt%, the change in thermal expansion rate is obvious. Beyond 5 wt% CNT, as the CNT content increases, the absolute value of the thermal expansion rate |*ε*| becomes smaller gradually. However, unlike the uni-directional nanocomposites (Figure 5), the thermal expansion rate of the multi-directional nanocomposites still decreases proportionally to the CNT content even when the CNT content is over 10 wt%.

### Verification

To verify the effectiveness of the above multi-scale numerical simulations, the following theoretical prediction and experimental measurements were carried out.

#### Theoretical prediction

The following assumptions are made to derive conventional micromechanics models for the coefficient of thermal expansion (CTE). Note that the CTE, which is generally understood as a constant and temperature-independent, is different from the thermal expansion rate used here. Following the terminology of conventional micromechanics models, we still use CTE in this section. The two-phase composite consisting of matrix and short fiber is of perfect interfaces at phase boundaries. Therefore, it is impossible for the two components, i.e., the matrix and short fiber, to separate at their interfaces when the composite is loaded or heated. Additionally, only macro-composites are considered, namely the scale of the reinforcement is large compared to that of the atom size or grain size so that composite properties can be modeled by continuum methods. This assumption may be reasonable here since the present MWCNT is comparatively large in diameter. Finally, the composite properties are an appropriate average of those of the components.

The CTE of a composite with short-fiber orientation distribution function *f*(*φ*), which is independent of dimension, can be given by [18]

(1) $$\begin{array}{c} \alpha_{c}=\frac{V_{f}E_{f}\alpha_{f}+V_{m}E_{m}\alpha_{m}}{V_{f}E_{f}+V_{m}E_{m}}\int_{0}^{x}(\cos^{2}\varphi -\nu_{c}\sin^{2}\varphi )f\left(\varphi \right)d\varphi \\ +\left[\left(1+\nu_{m}\right)\alpha_{m}V_{m}+\left(1+\nu_{f}\right)\alpha_{f}V_{f}\right]\int_{0}^{x}f\left(\varphi \right)d\varphi . \end{array}$$

For nanocomposites which contain a uni-directionally aligned reinforcement phase (e.g., MWCNT), *f*(*φ*) = 1, and therefore, the CTE of the nanocomposites is

(2) $$\alpha_{c}=\frac{V_{f}E_{f}\alpha_{f}+V_{m}E_{m}\alpha_{m}}{V_{f}E_{f}+V_{m}E_{m}}.$$

If MWCNTs are randomly orientated, the orientation distribution function *f*(*φ*) = 1/*n*, where *n* represents the number of different orientations of the MWCNTs in the matrix. If *n* is the number of possible orientations, the CTE of the nanocomposites is

(3) $$\begin{array}{l} \alpha_{c}=\frac{1}{2}\left(\frac{V_{f}E_{f}\alpha_{f}+V_{m}E_{m}\alpha_{m}}{V_{f}E_{f}+V_{m}E_{m}}\left(1-\nu_{c}\right)\right)\\ \left(\;+\left(1+\nu_{m}\right)\alpha_{m}V_{m}+\left(1+\nu_{f}\right)\alpha_{f}V_{f}\right). \end{array}$$

In the above equations, the nomenclatures for the parameters are as follows:

α, CTE

V, volume fraction

E, Young's modulus

ν, Poisson's ratio

and the subscripts are as follows:

c, nanocomposite

m, the matrix

f, the reinforcement phase (MWCNT here)

Note that Poisson's ratio of the nanocomposites, *v*_c_ in Equation 3, was directly obtained from the rule of mixture and the data in Table 2. For 1 ~ 5 wt% addition of CNTs, *v*_c_ ranges from 0.338 (1 wt%) to 0.333 (5 wt%).

#### Experimental measurements

**Figure 8:**
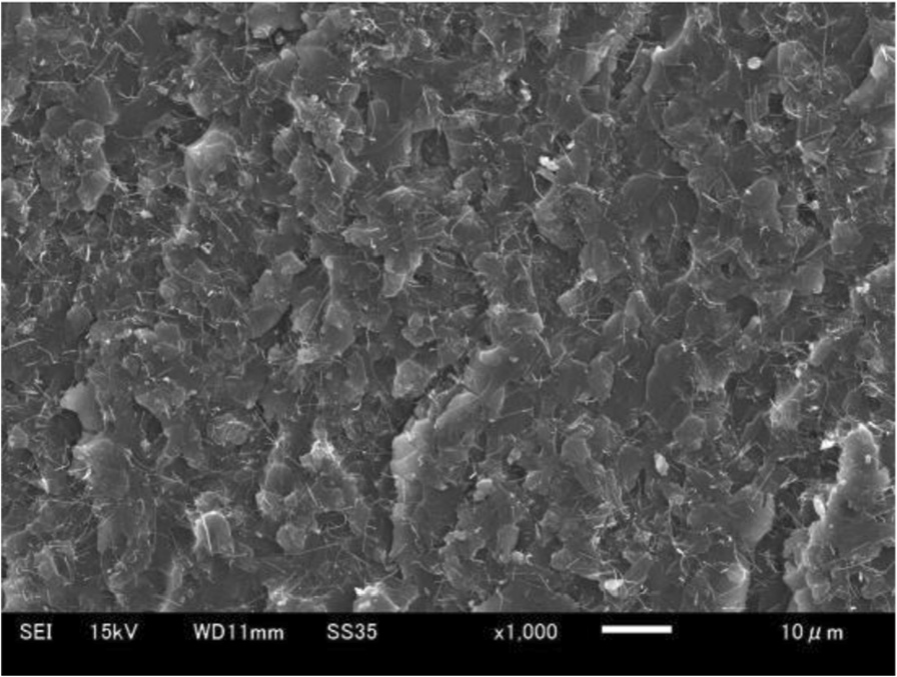
Dispersion state of MWCNT in epoxy matrix (3 wt%).

In the present experiments, MWCNTs were made via chemical vapor deposition, with purity above 99.5% (Hodogaya Chemical Co., Ltd., Tokyo, Japan). The detailed data have been listed in Tables 1 and 2. An insulating bisphenol-F epoxy resin (JER806, Japan Epoxy Resins Co., Ltd., Tokyo, Japan) and an amine hardener (Tomaido 245-LP, Fuji Kasei Kogyo Co., Ltd., Osaka, Japan) were used as matrix. The MWCNT/epoxy nanocomposites were prepared by mixing the epoxy and the hardener using a planetary mixer (AR-100, THINKY Co., Ltd., Tokyo, Japan) at 2,000 rpm for 30 s. Then, the MWCNTs were added into the mixture and mixed again at 2,000 rpm for 10 min. The final mixture was poured into a silicon mold and cured in a vacuum oven at 80°C for 2 h. This nanocomposite fabrication method was the same with that in the authors' previous experimental work [19-21], in which very good dispersion states of the MWCNTs under 3 and 5 wt% loading were identified (see image from scanning electron microscope observation in Figure 8 for the fractured surface of a 3 wt% sample).

The thermal expansion properties of the MWCNT/epoxy nanocomposites were measured using a TMA equipment (TMA-50, Shimadzu Co., Kyoto, Japan). The TMA measurement methodology is described as follows: a rectangular sample (3 cm wide, 3 cm long) was cut from the nanocomposites at a point 3 cm from the parallel portion of the tensile test specimen (according to JIS K 7197 [22]). Specimens were heated from 30°C to 120°C at a scanning rate of 5°C/min in air for continuous measurements. The thermal expansion properties of pure epoxy were similarly measured for the same specimen size and test conditions. Note that the highest test temperature, i.e., 120°C, is close to the glass transition point of bisphenol-F epoxy resin, which usually ranges from 120°C to 130°C, depending on fabrication conditions. In our tests, it was found that even at 120°C, the obtained thermal expansion rates were still normal and a molten or rubber-like state in epoxy was not identified.

#### Comparison

**Figure 9:**
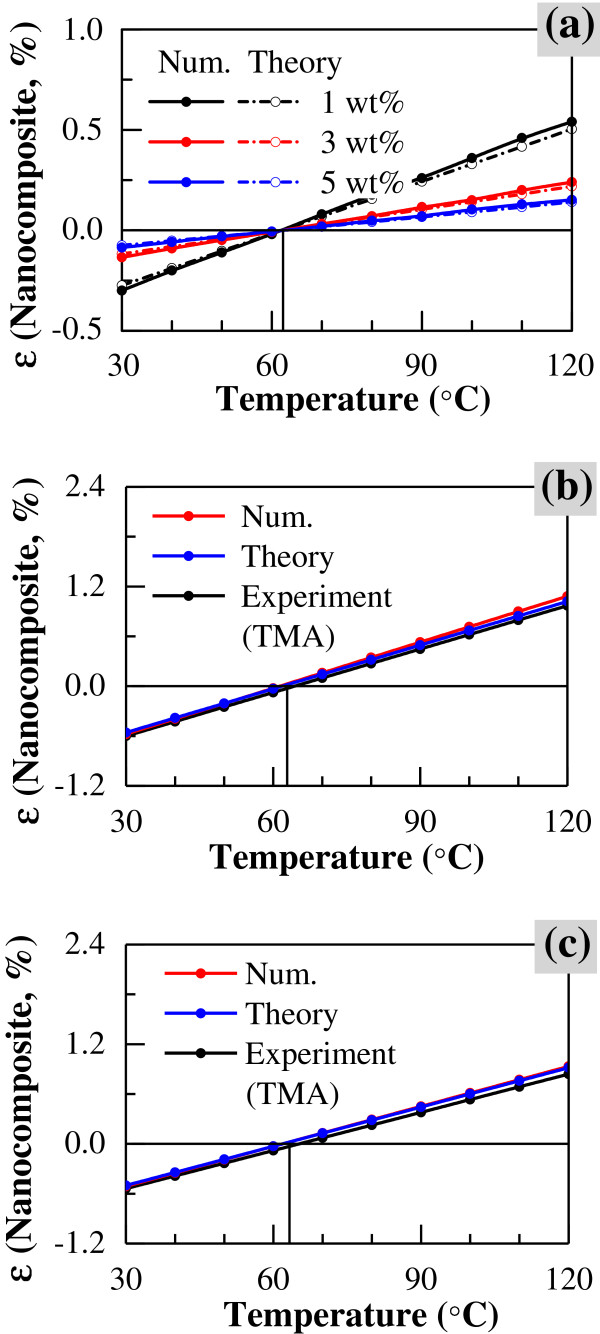
**Comparison of experimental, numerical, and theoretical results. **(**a**) Simulated and theoretical results (uni-directional CNT/epoxy nanocomposite), (**b**) experimental, simulated, and theoretical results for 1 wt% (multi-directional CNT/epoxy nanocomposite), (**c**) experimental, simulated, and theoretical results for 3 wt% (multi-directional CNT/epoxy nanocomposite).

**Figure 10:**
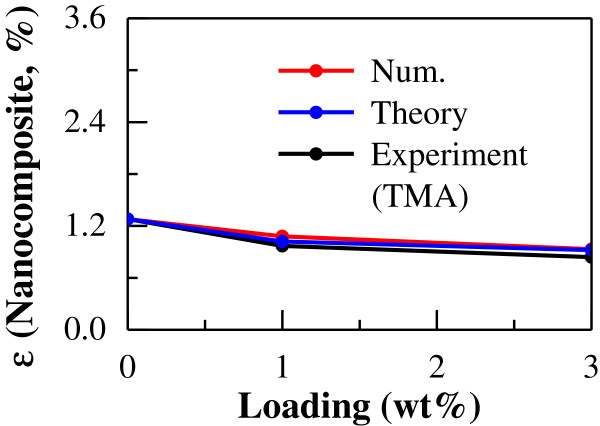
Relationship between CNT content and thermal expansion rate of CNT/epoxy nanocomposite at 120°C.

Figure 9 shows the comparison between the thermal expansion properties of the MWCNT/epoxy nanocomposites as determined by multi-scale numerical simulations, theoretical analysis, and experimental measurement. In Figure 9a, for uni-directional models, the comparison between the thermal expansion properties by multi-scale numerical simulation and theoretical prediction was given, in which the relative difference is lower than 15% for the results. In Figure 9b,c, for multi-directional models, the comparisons of experimental, simulated, and theoretical results were shown for different CNT contents (i.e., 1 and 3 wt%). It can be found that the multi-scale numerical simulation results possess a similar trend to the theoretical prediction and experimental measurement as temperature increases. It should be noted that the relative difference is also lower than 15% for all three results. This implies that the present multi-scale numerical simulation is effective in predicting the thermal expansion properties of CNT-based nanocomposites under the condition that the CNT is of a comparatively large size and a good dispersion state in matrix. Figure 10 shows the influence of CNT loading on the thermal expansion rates of the MWCNT/epoxy nanocomposites at high temperature (120°C), which was evaluated by experimental, simulated, and theoretical approaches. From this figure, it can be found that the thermal expansion rate obtained by experiments decreases about 25% at 1 wt% and 35% at 3 wt%. Moreover, a similar trend is observed at a broad temperature range from 30°C to 120°C, in which the thermal expansion rate decreases with CNT loading for each case, and the present numerical simulation and theoretical analysis can effectively predict the experimental measurements. This indicates that the addition of CNT leads to a considerable reduction in the thermal expansion rate of the MWCNT/epoxy nanocomposites, where CNTs with comparatively large size are well dispersed in the matrix in the present study. This characteristic leads to some special potential applications, such as good dispersion of CNTs into the matrix of carbon fiber-reinforced plastic to reduce residual stresses induced in the fabrication process. However, in many practical experiments, both distribution and dispersion of the CNTs may be nonuniform because of the different properties of CNTs and fabrication methods; practical agglomeration of CNTs in the matrix may weaken this positive effect, i.e., reduction of the thermal expansion rate of the matrix.

## Conclusions

In this work, the thermal expansion properties of CNT/epoxy nanocomposites with CNT content ranging from 1 to 15 wt% were investigated using a multi-scale numerical technique in which the effects of two parameters, temperature and CNT content, were investigated extensively. For all CNT contents, the obtained results clearly revealed that within a wide low-temperature range (30°C ~ 62°C), the nanocomposites undergo thermal contraction, and thermal expansion appears in a high-temperature range (62°C ~ 120°C). It was found that at any CNT content, the thermal expansion properties vary with the temperature. As temperature increases, the thermal expansion rate increases linearly. However, at a specified temperature, the absolute value of the thermal expansion rate decreases nonlinearly as the CNT content increases. Moreover, the results provided by the present multi-scale numerical model are verified with those obtained from a micromechanics-based theoretical model and from experimental measurement. Therefore, this multi-scale numerical approach is effective to evaluate the thermal expansion properties of any type of CNT/polymer nanocomposites.

## Competing interests

The authors declare that they have no competing interests.

## Authors’ contributions

Alamusi performed the numerical simulations, theoretical analysis, and experiment. NH, JQ, and YL designed the concept, analyzed the results, and drafted, revised, and finalized the manuscript with partial contribution of CC, SA, HF, YL, HN, LW, JL, WY, TW, CY, and YZ. All authors read and approved the final manuscript.

## References

[B1] Haggenmueller R Guthy C Lukes JR Fischer JE Winey KI Single wall carbon nanotube/polyethylene nanocomposites: thermal and electrical conductivity Macromolecules 2007 40 2417 2421 10.1021/ma0615046

[B2] Biercuk MJ Llaguno MC Radosavljevic M Hyun JK Johnson AT Fischer JE Carbon nanotube composites for thermal management Appl Phys Lett 2002 80 2767 2769 10.1063/1.1469696

[B3] Ruoff RS Lorents DC Mechanical and thermal properties of carbon nanotubes Carbon 1995 33 925 930 10.1016/0008-6223(95)00021-5

[B4] Hu N Karube Y Cheng Y Masuda Z Fukunaga H Tunneling effect in a polymer/carbon nanotube nanocomposite strain sensor Acta Mater 2008 56 2929 2936 10.1016/j.actamat.2008.02.030

[B5] Hu N Karube Y Arai M Watanabe T Yan C Li Y Liu Y Fukunaga H Investigation on sensitivity of a polymer/carbon nanotube composite strain sensor Carbon 2010 48 680 687 10.1016/j.carbon.2009.10.012

[B6] Seidel GD Stephens SN Analytical and computational micromechanics analysis of the effects of interphase regions on the effective coefficient of thermal expansion of carbon nanotube-polymer nanocomposites Proceedings of the 51st AIAA/ASME/ASCE/AHS/ASC Structures, Structural Dynamics, and Materials Conference: April 12–15 2010; Orlando 2010 Reston: AIAA 2010 2809

[B7] Wei C Thermal expansion and diffusion coefficients of carbon nanotube-polymer composites Nano Lett 2002 2 647 650 10.1021/nl025554+

[B8] Hu N Fukunaga H Lu C Kameyama M Yan B Prediction of elastic properties of carbon nanotube-reinforced composites Proc R Soc Lond A Math Phys Sci 2005 461 1685 1710 10.1098/rspa.2004.1422

[B9] Hu B Hu N Li Y Akagi K Yuan W Watanabe T Cai Y Multi-scale numerical simulations on piezoresistivity of CNT/polymer nanocomposites Nanoscale Res Lett 2012 7 402 10.1186/1556-276X-7-402 22804919 PMC3441497

[B10] Clancy TC Frankland SJV Hinkley JA Gates TS Multiscale modeling of thermal conductivity of polymer/carbon nanocomposites Int J Therm Sci 2010 49 1555 1560 10.1016/j.ijthermalsci.2010.05.007

[B11] Park C Wilkinson J Banda S Ounaies Z Wise KE Sauti G Lillehei PT Harrison JS Aligned single wall carbon nanotube polymer composites using an electric field J Polym Sci, Part B: Polym Phys 2006 44 1751 1762 10.1002/polb.20823

[B12] Okabe T Motani T Nishikawa M Hashimoto M Numerical simulation of microscopic damage and strength of fiber-reinforced plastic composites Adv Compos Mater 2012 21 147 163 10.1080/09243046.2012.688495

[B13] Huang H Talreja R Numerical simulation of matrix micro-cracking in short fiber reinforced polymer composites: initiation and propagation Compos Sci Technol 2006 66 2743 2757 10.1016/j.compscitech.2006.03.013

[B14] Alamusi Hu N Jia B Arai M Yan C Li J Liu Y Atobe S Fukunaga H Prediction of thermal expansion properties of carbon nanotubes using molecular dynamics simulations Comput Mater Sci 2012 54 249 254

[B15] Yamamoto G Liu S Hu N Hashida T Liu Y Yan C Li Y Cui H Ning H Wu L Prediction of pull-out force of multi-walled carbon nanotube (MWCNT) in sword-in-sheath mode Comput Mater Sci 2012 60 607 612

[B16] Hu N Fukunaga H Lu C Kameyama M Yan B Prediction of elastic properties of carbon nanotube-reinforced composites Proc R Soc A 2005 461 1685 1710 10.1098/rspa.2004.1422

[B17] Hu N Wang B Tan GW Yao ZH Yuan W Effective elastic properties of 2-D solids with circular holes: numerical simulations Compos Sci Technol 2000 60 1811 1823 10.1016/S0266-3538(00)00054-3

[B18] Wang YQ Zhang MD Zhou BL Shi CX A theoretical model of composite thermal expansion Mater Sci Prog 1989 3 442 446

[B19] Hu N Masuda Z Yamamoto G Fukunaga H Hashida T Qiu J Effect of fabrication process on electrical properties of polymer/multi-wall carbon nanotube nanocomposites Composites: Part A 2008 39 893 903 10.1016/j.compositesa.2008.01.002

[B20] Hu N Li Y Nakamura T Katsumata T Koshikawa T Arai M Reinforcement effects of MWCNT and VGCF in bulk composites and interlayer of CFRP laminates Composites: Part B 2012 2012 43 3 9

[B21] Li Y Hu N Kojima T Itoi T Watanabe T Nakamura T Takizawa N Inoue T Cui H Atobe S Fukunaga H Experimental study on mechanical properties of epoxy/MWCNT nanocomposites - effects of acid treatment, pressured curing, and liquid rubber ASME J Nanotechnol Eng Med 2012 3 011004 10.1115/1.4007018

[B22] Japanese Industrial Standards Committee JIS K 7197–1991: Testing Method for Linear Thermal Expansion Coefficient of Plastics by Thermomechanical Analysis 1991 Tokyo

